# Historical, archaeological and linguistic evidence test the phylogenetic inference of Viking-Age plant use

**DOI:** 10.1098/rstb.2020.0086

**Published:** 2021-07-05

**Authors:** Irene Teixidor-Toneu, Anneleen Kool, Simon J. Greenhill, Karoline Kjesrud, Jade J. Sandstedt, Vincent Manzanilla, Fiona M. Jordan

**Affiliations:** ^1^ Natural History Museum, University of Oslo, Sars' Gate 1, 0562 Oslo, Norway; ^2^ Naturalis Biodiversity Center, Darwinweg 2, 2333CR Leiden, The Netherlands; ^3^ Australian National University College of Arts and Social Sciences, ARC Centre of Excellence for the Dynamics of Language, Canberra, ACT 2601, Australia; ^4^ Department of Linguistic and Cultural Evolution, Max-Planck-Institut fur Menschheitsgeschichte, 07745 Jena, Thüringen, Germany; ^5^ Museum of Cultural History, University of Oslo, St Olavs Gate 29, 0130 Oslo, Norway; ^6^ BaseClear, Sylviusweg 74, 2333BE Leiden, The Netherlands; ^7^ Department of Anthropology and Archaeology, University of Bristol, Bristol BS8 1UU, UK

**Keywords:** cultural evolution, ethnobotany, interdisciplinary research, phylogenetic comparative methods

## Abstract

In this paper, past plant knowledge serves as a case study to highlight the promise and challenges of interdisciplinary data collection and interpretation in cultural evolution. Plants are central to human life and yet, apart from the role of major crops, people–plant relations have been marginal to the study of culture. Archaeological, linguistic, and historical evidence are often limited when it comes to studying the past role of plants. This is the case in the Nordic countries, where extensive collections of various plant use records are absent until the 1700s. Here, we test if relatively recent ethnobotanical data can be used to trace back ancient plant knowledge in the Nordic countries. Phylogenetic inferences of ancestral states are evaluated against historical, linguistic, and archaeobotanical evidence. The exercise allows us to discuss the opportunities and shortcomings of using phylogenetic comparative methods to study past botanical knowledge. We propose a ‘triangulation method’ that not only combines multiple lines of evidence, but also quantitative and qualitative approaches.

This article is part of the theme issue ‘Foundations of cultural evolution’.

## 1. Introduction

Humans are exceptional at spreading knowledge, materials and technologies across space and time. Identifying the processes of transmission and accumulation of cultural traits is an underlying goal of the humanities and social sciences; one of the grand challenges of the new field of cultural evolution [1]. Disciplines differ in the scales at which these processes are considered, and they can range from ‘micro-evolutionary’ studies of historical texts [2] to ‘macro-evolutionary’ studies of social norms across societies [3]. Cultural evolutionary scholars view the coherent scaling up and down between these levels to be a desirable goal with the potential to integrate knowledge on shared topics across disciplines. Collaborative work in recent years has begun to produce large-scale cross-cultural datasets of population-level data on cultural and social practices and relevant ecological variables (e.g. [4], see [5]). Moreover, phylogenetic comparative methods (PCMs; see e.g. [6–9]) are now widely used, both to control for historical autocorrelation and to model macro-level processes of transmission between populations.

The task of linking group-level, deep-time dynamics with the specifics of contemporary ethnographic or behavioural accounts of cultural transmission (or even the mathematical modelling of social learning) remains a key challenge [10]. Phylogenetic approaches to culture are often focused on ‘big picture’ questions [8] about the evolution of e.g. political systems [11], religious beliefs [12] or social structure [13] across multiple millennia; these grand narratives are not easily connected to the specifics of intra-population cultural transmission. To facilitate this connection, we take a ‘meso-evolutionary’ approach [14] that we characterize as (i) multidisciplinary and mutually reinforcing with respect to data sources and methods, (ii) focused on data-rich case studies in a restricted domain, culture area/language family and timespan, thus ensuring the emic comparability and historical connections of the cultural phenomena and (iii) generating cultural patterns from the bottom up where possible, rather than imposing external categories. The results of such investigations will inform, be informed by, and ultimately bridge micro- and macro-evolutionary studies of culture.

Here, we focus on the botanical knowledge of Nordic populations speaking North Germanic languages. The North Germanic or ‘Nordic’ languages form a subgroup of Germanic languages, spoken in Northern Europe. North Germanic people emerged as a linguistically and culturally distinct group in the early centuries CE. Old Norse, the ancestral language to modern Nordic varieties, was spread to Iceland, the Faroe Islands and Shetland around 800 CE. During the Old Norse period, all Nordic varieties were mutually intelligible and are traditionally divided into two main dialectal groups: Old West Norse (Old Icelandic, Old Faroese, Old Norn and Old Norwegian) and Old East Norse (Old Swedish, Old Danish and Old Gutnish [15,16]). The earliest common ancestor to all modern Nordic languages can thus be traced back to the Viking-Age (Early Old Norse, *ca* 700–1000 CE). With the introduction of Christianity around the year 1000 and the ruler ideology that followed, the former itinerant kingships were merged and power distribution centralized within larger geographical areas. The power institutions influenced both material and written culture, and contributed to a gradual standardization of the vernacular languages in the overall dialectal patterns [15,16].

Plants are a prerequisite for human life and culture. Materially, they provide health, food, shelter and technology; plants also convey cultural meaning and can symbolize group identities. Studying past uses is key to understanding the role of plants for past human populations but is impeded by three challenges. First, archaeobotanical remains are limited, and their interpretation is difficult. Second, pre-modern plant knowledge was most often transmitted orally; early written historical sources mentioning plants are sparse, and written vernacular names can be difficult to link to taxonomic species. Third, while historical linguistic analysis can add to our knowledge of past plant importance and uses, inferences from vernacular names are not necessarily possible for all plants. This knowledge gap is especially evident for the Viking-Age.

Archaeobotanical remains of at least 100 plants with potential (though not always confirmed) uses for food, brewing, medicine, textiles, dyes and building materials have been recovered from Viking-Age sites (e.g. [17–19]). However, archaeobotanical interpretations typically focus on general plant ecology; on what can be inferred about the landscape around a settlement or on *potential* use based on current plant traditions rather than use-related archaeological context and contemporary cross-disciplinary sources (e.g. [20]).

The earliest written vernacular plant names are found in runic inscriptions from the fifth to sixth century, but there is extremely limited documentation of plant use in Viking-Age runic inscriptions and literary sources (poetry). The thirteenth century provides the first detailed descriptions of plant use in medieval Scandinavia (importantly evident in the first Nordic herbals), and the descriptions of plants in a variety of medieval sources testify to both practical and symbolic plant value. Plant names, including loaned vocabulary, can provide tangible known limits for dating plant knowledge where we know the chronology of language contact. For example, Sw. *färgare veide* ‘dyeing-isatis plant’ (woad, *Isatis tinctoria*) is loaned from the post-Viking-Age Middle Low German word *verwe* ‘colour, dye’ and does not provide direct linguistic evidence of Viking-Age use in dyeing. By contrast, the phonological form of the North Sámi (descendant of Proto-Sámi, neighbouring Uralic group) word for sorrel (*Rumex acetosa*), *suvrrarássi* ‘sour-grass’, is loaned from Proto-Norse and preserves pre-Viking-Age Proto-Norse unstressed vowels, confirming the early consumption of sorrel in Norse-speaking populations. Other vernacular plant names that use native Nordic vocabulary typically cannot be dated by linguistic evidence alone as the names could equally likely be old or more recent.

**Figure 1.**
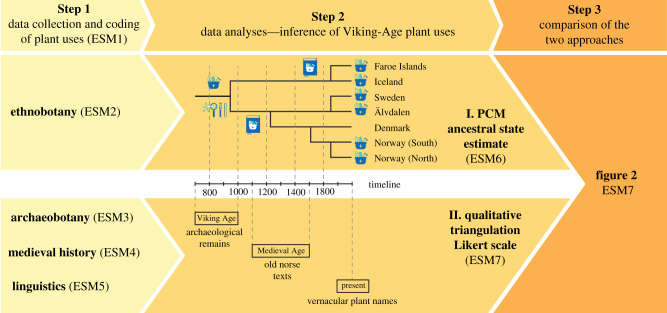
Conceptual framework and methodological step-by-step comparison of estimated plant uses in the Viking-Age. Each step is linked to the relevant figures and electronic supplementary material (ESM). Dating of archaeological evidence, medieval texts, linguistic and ethnobotanical evidence is indicated on the timeline of Step 2. (Online version in colour.)

We test the utility of PCMs to infer Viking-Age plant use. We combine multiple datasets of historical, linguistic and archaeological data to cross-validate computational evolutionary modelling based on ethnographic data [21] (figure 1). We hypothesize that some plant-use knowledge is transmitted vertically across time and can be traced back to the Viking-Age. To test this proposition, we use a language tree to model population history and computationally infer ancestral states using PCMs from recent ethnographic evidence. We then compare the model's results against our other data sources. We expect that ancestral plant uses (i) will be documented to some extent in the medieval historical record, (ii) might be referred to by plant vernacular names and (iii) that archaeobotanical remains for these plants will have been found in Viking-Age sites. Our research questions fall within the scope of relatively shallow time depths, while still extending beyond the ethnographic present. Ethnographic data are often used in non-systematic ways to provide insights into pre-historic culture; here we aim to bring together rich data and expertize on the historically and geographically bounded cultural sphere of Viking-Age and medieval Norse populations to scrutinize phylogenetic approaches in the light of direct archaeological and historical evidence, further enabling cross-disciplinary communication [22]. This provides a unique opportunity to investigate in what ways ancestral state inference using ethnobotanical data complements the archaeological, historical and linguistic evidence.

## 2. Methods

Twelve plant species were chosen to capture four phenomena: plants widely and not-so-widely used across Scandinavia; plants with native distributions across the Nordic region or limited distributions; and plants present and absent from the historical and archaeobotanical Viking-Age record (table 1). Some archaeobotanical remains were interpreted as used or as surrounding vegetation by archaeobotanists in the literature after the first excavation. Most plants in the historical record had documented uses, but not all. Plant uses were coded in general (e.g. medicinal) and specific (e.g. respiratory) categories, following economic botany standards (electronic supplementary material, S1; figure 1, Step 1). General plant uses are not a strict addition of specific uses, as they also include unspecified information (e.g. ‘improves health’ would be scored as medicinal but not as any specific use). We consider Viking-Age plant uses to be those from Early Old Norse-speaking populations (*ca* 700–1000 CE, hereafter Old Norse; figure 1).

**Table 1.** The evidence base of the plant species selected for this study. All plants are geographically distributed across the Nordic countries except for those marked (*), which are not present in Iceland or the Faroe Islands. Use acronyms refer to: Agri, agricultural; AnFood, animal food; Constr, construction; Food, Fue; IndCraft, industry and crafts; Med, medicine; SSR, social, symbolic and ritual; Vet, Veterinary. Vernacular names may indicate use, but might be later loans or only present in one or few of the North Germanic languages, hence not necessarily providing evidence of presence for the Viking-Age. (ON) in the Linguistics column indicates that there is a name that can be traced back to Old Norse.

plant	archaeobotany (Viking-Age)	medieval sources (Old Norse)	linguistics (modern)	ethnobotany (modern)
*Achillea millefolium* L. (yarrow)	present	Med, SSR	Food, IndCraft, Med (ON)	Food, IndCraft, Med, SSR, Vet
*Angelica archangelica* L. (angelica)	present	Agri, IndCraft, SSR	Food, IndCraft (ON)	AnFood, Food, IndCraft, Med, SSR, Vet
*Cornus suecica* L. (bunchberry)	present [Food]	—	AnFood, Med (ON)	AnFood, Food, Med, SSR
*Euonymus europaeus* L. (spindle)*	present [IndCraft]	present	IndCraft, Med	Constr, Fuel, IndCraft, Med, SSR, Vet
*Filipendula ulmaria* (L.) Maxim. (meadowsweet)	present [AnFood]	Agri, Med	Food	Agri, AnFood, Food, IndCraft, Med, SRR, Vet
*Humulus lupulus* L. (hops)*	present [Food, IndCraft]	Agri, Food, Med	(ON)	Agri, AnFood, Food, Fuel, IndCraft, Med, SRR, Vet
*Isatis tinctoria* L. (woad)*	present [IndCraft]	Med	IndCraft	IndCraft
*Juniperus communis* L. (juniper)	present [Constr]	Agri, Fuel, Med	Fuel, IndCraft (ON)	Agri, AnFood, Constr, Food, Fuel, IndCraft, Med, SRR, Vet
*Knautia arvensis* (L.) Coult. (field scabious)	present	—	Food, Med	Agri, IndCraft, Med, SSR
*Rhodiola rosea* L. (roseroot)	—	—	Med (ON)	AnFood, Food, IndCraft, Med, SSR, Vet
*Rumex acetosa* L. (sorrel)	present	Food, Med, SSR	Food (ON)	AnFood, Food, IndCraft, Med, SSR, Vet
*Stellaria media* (L.) Vill. (chickweed)	present	Med	Agri, AnFood, Food, IndCraft, Med (ON)	AnFood, Food, IndCraft, Med

### (a) Ethnobotanical data collection, tree and ancestral state estimation

Ethnobotanical data were collected for the 12 selected plants in six extant North Germanic languages (Norwegian, Danish, Swedish, Elfdalian, Icelandic and Faroese). Plant-use combinations were coded for each language from a combination of twentieth-century national ethnobotany compilations and eighteenth-century floras (electronic supplementary material, S2). Some uses were not well represented by any code and were arbitrarily categorized as ‘other’; we did not consider those further in making past use inferences. Plant-use combinations not present across ethnobotanical sources were coded as 0, except in the case of Elfdalian for which sources are extremely partial and thus absences were coded as NA. This conservative approach does not over-infer presence when we cannot rule out that a plant did not have a particular use. Norwegian ethnobotanical data were localized, so we distinguished a northern and a southern dataset to add power to our analyses.

These ethnographic data were used to infer ancestral states for plant-use combinations. A representative posterior sample of 1000 North Germanic language trees were pruned from a larger Indo-European phylogeny built with basic vocabulary data [23] (figure 1, Step 2.I). To incorporate both the northern and southern trait clusters in the Norwegian branch, we split the Norwegian language branch in two using the treeman package [24]. We created an R package, noRdic, to load and manipulate data into the format needed for ancestral state inference (https://github.com/SimonGreenhill/noRdic). The function rayDISC in the corHMM R package was used to estimate ancestral states [25]. Due to the small size of the tree, we estimated the ancestral states under the simplest model of equal transition rates for the gain and loss of each trait. We corrected for ascertainment bias following Lewis [26], and we specified the prior root probability to be equal across states.

### (b) Archaeobotanical, historical and linguistic data collection and analyses

Archaeobotanical data were obtained from the published literature on finds at Viking-Age sites in Scandinavia, Northern Germany, the British Isles, the Faroe Islands, Russia and Greenland. We documented potential use from published archaeological interpretations if they were based on context (electronic supplementary material, S3). We focussed the data collection primarily on macrofossil finds; pollen data that rendered information on use or human migration were also included.

Old Norse plant uses were traced in primarily medieval Old West Norse literature covering charters, biblical texts, sermons, legal texts, encyclopedic works and saga literature, following Heizmann [27]. In line with New-Philology strategies [28], we cross-checked with the actual mentions in medieval manuscripts digitally available through the Dictionary of Old Norse Prose [29]. The entire corpus of West Old Norse Literature from the twelfth to sixteenth century was covered, with the exception of poetic literature. Each mention of a plant was registered together with its corresponding use (electronic supplementary material, S4). Old Swedish plant uses have been traced in Old Swedish medicinal books from the fifteenth to seventeenth century, available in philological editions from the nineteenth century, later collated by Larsson [30].

Vernacular plant names were collected from the same sources as ethnobotanical data (electronic supplementary material, S5). Plant name data were encoded at three linguistic stages: (i) each plant's vernacular name(s) (e.g. Norwegian *kvann*, Icelandic *hvönn*, North Sámi *fádnu* for angelica (*Angelica archangelica*)), (ii) each name's corresponding source words, encoded as their earliest written attestation (e.g. Old Norse *hvo¸nn*) and (iii) the earliest accepted reconstruction of the source word (e.g. Proto-Germanic *hwannō). Source words were translated and interpreted for potential uses indicated by the plant name (e.g. Swedish *älg-gräs* ‘ale-grass’ indicating use of meadowsweet (*Filipendula ulmaria*) in beer brewing). Old Norse and Proto-Germanic representations follow the Dictionary of Old Norse Prose [29] and Kroonen's [31] *Etymological dictionary of Proto-Germanic*, respectively, supplemented by reconstructions in wiktionary.org where necessary. Vernacular names are encoded per language family (e.g. North Germanic, Sámi, etc.) and historic/reconstructed source words are further categorized by source language. Using these three stages and linguistic groupings, we can capture and differentiate between historic and pre-historic linguistic borrowings in the data. When historically documented (or securely reconstructed) native vocabulary for a given species exists, we infer that it had a concrete and consistent use in the community.

Archaeologically or historically evidenced plant uses were coded in the same general and specific uses as for ethnobotanical data (electronic supplementary material, S1). Archaeobotanical, historical and linguistic data were assessed together qualitatively to infer potential Viking-Age plant uses following the ‘triangulation’ approach championed by Kirch & Green [32] (figure 1, Step 2.II). To combine these lines of evidence, potential Viking-Age plant uses were scored using a Likert scale (1 = no evidence, 2 = unlikely, 3 = possible but not confirmed, 4 = highly likely, 5 = confirmed evidence), with high scores (4 or 5) interpreted as evidence for Viking-Age plant use. When archaeobotanical finds provided evidence of use inferred from the archaeological context, these were scored as confirmed evidence of use; finds interpreted on the basis of later use of the plant were not considered as positive evidence. Historical evidence was scored more highly if mentions of use were present in unrelated sources. Linguistic evidence was scored more highly when source words could be traced to Early Old Norse. These inferences were then compared to the probabilities estimated through ancestral state estimation from ethnobotanical data (figure 1, Step 3).

### (c) Comparison and combination of PCM estimates with archaeological, historical and linguistic evidence to reconstruct Viking-Age plant use

Predictions from different qualitative and quantitative data are first tested against one another and then combined to reconstruct Viking-Age medicinal and food plant uses. We further draw on Kirch & Green's [32] conceptualization of a ‘method of triangulation’ as the bringing together of multiple lines of evidence in an historical, phylogenetic framework, to combine inferences made through the two approaches. From their original ideas, we combine inferences based on each of our multidisciplinary datasets, with a further triangulation step afforded by formal phylogenetic methods.

## 3. Results

### (a) Comparing PCM estimates with archaeological, historical and linguistic evidence

**Figure 2.**
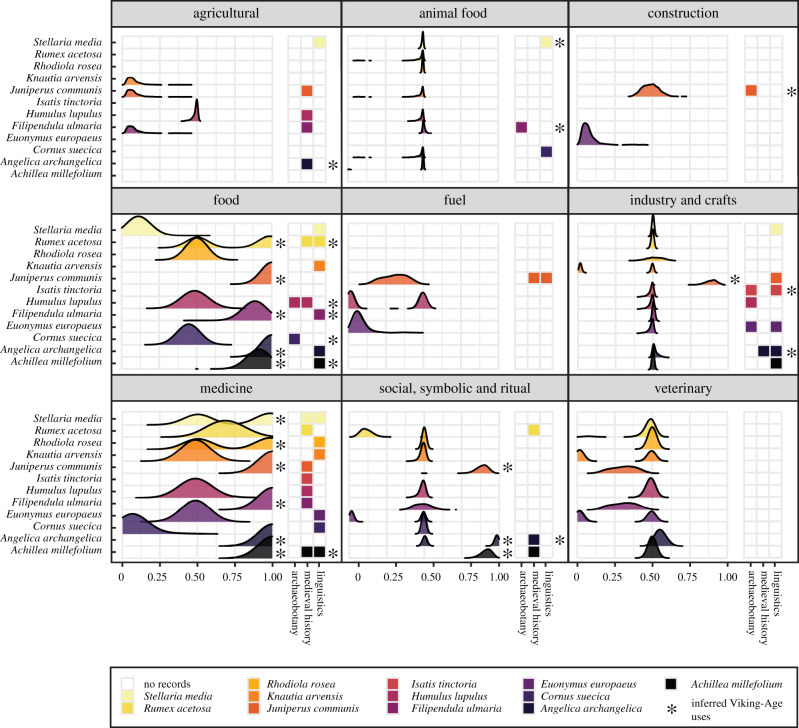
Combined evidence for each of nine general uses (panels) across 12 plant species (*y*-axis). Probability distributions indicate the presence of a trait in the root across 1000 phylogenies (*x*-axis); right-hand columns in each panel show presence of archaeological, historical and linguistic evidence. Highly likely Viking-Age uses as inferred from PCMs (mean probability of plant use being present at the root was equal to or higher than 0.75) or a triangulation of archaeological, historical and linguistic evidence (Likert score equal or higher than 4) are marked with*. (Online version in colour.)

We collected 348 general and specific plant-use traits from ethnobotanical sources for the 12 selected species. Using PCMs, a high probability of ancestral use was inferred for seven of the 12 plants (electronic supplementary material, S6, S7), i.e. their mean probability of being present at the root was equal to or higher than 0.75 (electronic supplementary material, S7; figure 2). Twenty-nine plant-use combinations were estimated ancestral with high probability; 15 were general (e.g. medicine, food) and 14 specific (e.g. digestive, respiratory). The bifurcating basal split of the tree could reasonably lead to root node ambiguity if a plant-use combination is present in one clade and absent in another. For the 40% of plant-use combinations with ancestral state estimate (ASE) between 0.45% and 0.55%, 31% are present in both basal clades, so we discard this as a primary cause of ambiguity.

Triangulating archaeobotanical, historical and linguistic data, we find evidence or infer high likelihood of use by Old Norse populations for nine of the 12 plants with 19 plant-use combinations, of which 13 are general uses and six are specific (electronic supplementary material, S7). When we compare the PCM-estimated and data-triangulated approaches, five general plant-use combinations and two specific combinations are observed as highly likely according to both approaches (figure 2).

All 12 plants selected were present in the Viking-Age archaeobotanical record with the exception of roseroot (*Rhodiola rosea*), which has very small seeds and a fleshy body that we would not expect to be preserved. However, only five plant uses can securely be deduced from the archaeological context (juniper (*Juniperus communis*) used to build fences, hops (*Humulus lupulus*) in beer brewing, woad as a dye, meadowsweet as hay and bunchberry (*Cornus suecica*) as edible; electronic supplementary material, S3). None of these use-combinations are estimated as highly likely by phylogenetic inference either because of the limited distribution of the plants (i.e. hops and woad) or the limited presence of these uses in the ethnobotanical record.

The medieval historical record (twelfth to sixteenth century) provided evidence of medicinal and ritual plant uses, cultivated plants (possibly for food purposes; electronic supplementary material, S4), foodstuffs and the symbolic value of some plants, but it is evidently non-exhaustive (e.g. there are no references to construction materials) and, in regard to medicine, heavily influenced by the Mediterranean *materia medica*. Of the 29 uses with high probability inferred through ancestral state estimation, 11 were described in medieval historical texts (electronic supplementary material, S7). This discrepancy mostly stems from the popularity of juniper in the modern ethnobotanical record. Juniper accounts for 11 of the 29 plant-use combinations inferred by PCM as ancestral, while there is little historical evidence for this diversity of uses: only three uses were confirmed by the medieval record. Moreover, the use of juniper as fuel is evidenced from the historical and linguistic records and yet not estimated as ancestral by PCMs. Historical evidence for consumption as food was generally low, and only found for two plants (hops and sorrel). When there was historical evidence for cultivation or harvest of a plant, or its use as a foodstuff (angelica, sorrel, juniper, hops; electronic supplementary material, S7), edible uses were inferred as ancestral by PCMs, with the exception of hops given their limited geographical distribution (figure 2). When a plant was estimated to be medicinal by PCM analyses (yarrow (*Achillea millefolium*), angelica, meadowsweet, juniper, roseroot, chickweed (*Stellaria media*); figure 2), evidence of medicinal use was found in the historical record, except for angelica and roseroot (electronic supplementary material, S7).

All plants for which ancestral uses were inferred had vernacular names with shared North Germanic word roots, confirming the past knowledge and importance of these species. Plants with no inferred ancestral use have either highly inconsistent naming traditions within and across the Nordic languages (e.g. field scabious (*Knautia arvensis*), electronic supplementary material, S5) or have geographical distributions limited to continental Scandinavia (e.g. hops and woad; table 1).

### (b) The ‘new’ triangulation method: combining PCM-estimated and data-triangulated approaches to reconstruct Viking-Age medicinal and food plant uses

Figure 2 showcases the potential of PCM estimates to complement archaeological, historical and linguistic evidence, and we summarize our findings per type of use in table 2.

**Table 2.** Summary of comparisons and triangulations to infer Viking-Age plant use.

type of use	Viking-Age plant use inference
agricultural	For the four species where data permitted inference, ancestral use was not inferred or was equivocal (*H. lupulus*; present in medieval texts). Historical texts and linguistic evidence did not overlap sufficiently with or support the PCM results. It may be that agricultural plant uses have evolved too rapidly due to technological change for triangulation approaches to be of high utility.
animal food	Eight plant species were inferred to have equivocal ancestral plant use, three of which were indirectly confirmed by linguistic or historical evidence.
construction	Only two species could be analysed with PCM. Results were equivocal for juniper (confirmed by archaeobotanical remains) and negative for spindle (no triangulated evidence).
food	This category neatly showcased the utility of the triangulation approach. Of nine species analysed, all but one of the eight positive or equivocal PCM results were corroborated/supported by the alternative evidence. PCMs contribute in adding clarity as to how plants were used as foodstuffs: use in alcoholic drinks (yarrow, juniper and meadowsweet) and as leaf vegetables (angelica and sorrel) are estimated ancestral where neither the archaeological nor the historical record provides such detail.
fuel	Similar to construction, PCM inferences of ancestral were negative to equivocal, again with juniper supported by linguistic and medieval sources.
industry and craft	Eleven species had sufficient data to infer material culture use by PCM. All were equivocal except juniper (present; supported by linguistics) and field scabious (absent; not contradicted by any other evidence). This category was one where the alternative lines of evidence were particularly valuable in shifting ambiguous PCM results to a more positive stance on Viking-Age presence.
medicine	Like food, this category was well served by triangulation. Almost all species had historical or linguistic evidence in support of their ancestral medicinal use as well as equivocal or positive PCM support. Linguistic and PCM evidence conflicted only for bunchberry. Again as in food, PCMs estimate some specific medicinal uses. Here though, some of these estimates should be considered carefully, given the spread of Mediterranean *materia medica* horizontally across the Nordic region later in time. This might be the case for yarrow's uses to treat injuries and digestive problems. Juniper is also a well-known medicinal plant in the European *materia medica*, yet respiratory and urological uses are absent from the medieval Nordic herbals (electronic supplementary material, S4) and their estimation through PCMs could hint towards a local Nordic medicinal tradition.
social, symbolic and ritual	Social, symbolic and ritual uses can benefit from a triangulation approach. PCMs provided evidence for the social value of three species, two of which were confirmed by the historical record.
veterinary	No archaeological, historical or linguistic evidence for veterinary uses is observed and all PCM inferences are equivocal. Rather than indicating a lack of veterinary medicine among past populations, this probably hints at biases in the source material where these kinds of uses were not thoroughly documented.

Considering general uses, we can make the two preliminary suggestions: (i) that the data-rich domains of food and medicine were well served by our approach and (ii) that equivocal PCM results may be obtained for domains where technological change may shift uses rapidly (industry and craft, agriculture, animal food). This latter set of uses may require greater reliance on triangulated evidence. Importantly, food and medicinal uses are more challenging to interpret from the archaeological context than industry and craft, agriculture and animal food uses. These results highlight the potential of PCM-based approaches to reconstruct past plant-related cultural traits.

## 4. Discussion

### (a) Inferring cultural evolution from multiple lines of evidence

From the starting point that culture is variably transmitted information, we explored a triangulation approach to ancestral plant use in the Nordic region. PCM results are both partly confirmed by linguistic, historical and/or archaeological data, and partly provide new hypotheses for the past uses of plants where archaeobotanical, historical and linguistic data do not suggest any particular use, or are inconclusive (figure 2; electronic supplementary material, S7). The archaeobotanical, historical and linguistic records are either partial or provide indirect evidence of Viking-Age plant use. The archaeological record is the only one able to provide direct evidence of plant use in the Viking-Age given the lack of written Viking-Age sources describing plant uses. As we observe here, plant use can rarely be inferred from the archaeological context in which macrofossils are found, and there is not necessarily a relation between how often macrofossils of a certain taxa are found and the potential for use. This is due to the diversity of morphological characteristics that are or are not preserved (or identified) and differences in the plant parts being used and how the plants were processed [33]. Onions and angelica are, for example, assumed to have been widely used during the Viking-Age, but leave few archaeobotanical remains because people used the vegative parts [34,35]. Yet PCMs infer ancestral medicinal and food uses of angelica among Old Norse-speaking populations. Angelica is present in the Viking-Age archaeobotanical record with undetermined uses, and mentions of this plant in medieval historical documents only refer to its cultivation, symbolic value or use as a tool. We interpret these kinds of overlaps as positive corroborative evidence.

PCMs also infer the ancestral use of meadowsweet and yarrow in alcoholic drinks (e.g. beer or mead brewing, figure 2). These uses are not documented in historical literature, but are evidenced in vernacular names (electronic supplementary material, S7). PCMs highlight the importance of plants (e.g. angelica) and uses (e.g. food) that do not feature prominently in the Nordic historical record, fuelling future research. This supports the potential of this approach [21].

We more often estimate general ancestral uses (e.g. medicine) than specific ones (e.g. to treat digestive issues; figure 2); this trend is also observed at shallower historical depths (eighteenth to twentieth century) [34]. If a plant is good as food, fodder or for construction depends to a large extent on its physical qualities. However, use as a spice or in desserts, as hay or in pastures, for building houses or boats, is much more variable between and within cultural groups even at these small geographical scales. The limited literature using PCMs on botanical data has thus far shown that plant knowledge for subsistence or medicine is not well predicted by cultural phylogeny [3,36]. This is likely due to both ecological constraints and the plasticity of plant traditions. As material technologies, plant uses may have fewer learning steps to become entrenched by cumulative cultural evolution [37,38]; their ubiquity may facilitate low-cost random copying [39]; and some technologies may show fewer intergenerational constraints than other cultural practices and norms [40]. However, PCMs are able to reconstruct some specific uses back to Old Norse (electronic supplementary material, S7), adding potential detail to our knowledge of how plants were used in the Viking-Age.

Botanical knowledge presents a paradox for models of cultural evolution. It can be quickly transformed and easily borrowed, while not widely spread cross-culturally or even between the individuals of a single culture. Yet some knowledge of plants, often both accessible and attractive, persists through time [41]. As frequently used words tend to be stable over time [42], native Old Norse word sources for plant names shared across the North Germanic languages may indicate plants that were commonly used throughout time. Taking this idea one step further, just as frequently used words persist, frequently used knowledge may also persist [41]. For example, our results show that flavouring alcoholic drinks is the specific use most often inferred to be ancestral in our dataset. PCMs infer that three out of four plants used in alcoholic drinks were used for this purpose already in the Viking-Age. A recent article highlights the cultural importance of ale for the Nordic peoples through time [43]. The plants added in alcoholic drinks are also conserved when we look at a historical time-lapse (eighteenth to twentieth century [34]). With our approach, we can identify the plant species that have kept their relevance over time.

### (b) Limitations to phylogenetic inference for the study of ‘meso-evolutionary’ processes

We infer ancestral states of plant use for North Germanic-speaking populations along a core-vocabulary phylogenetic tree [23]. Such trees are meant to capture historical relationships among the Nordic languages and can be used to infer shared traits transmitted via vertical inheritance (e.g. [4,21]). However, they do not represent all aspects of language history, masking, in particular, the effects of horizontal transmission via language contact [8,9,44]. For example, in the North Germanic clade, Norwegian would historically cluster with Icelandic and Faroese as West Nordic varieties, but due to later close contact with East Nordic languages, it has now diverged from insular Nordic languages and converged with neighbouring Scandinavian varieties, Danish and Swedish [45]. Although contact effects like the historical convergence of Norwegian with other Scandinavian varieties are not adequately captured by any single tree, trees remain the best available general model for most biological and historical linguistic relations [46]. By taking a Bayesian approach and calculating the fit over a posterior probability distribution of trees rather than a single tree, any uncertainty in the tree topology caused by horizontal transmission will be partially incorporated into the trees in the posterior, and our results will include this uncertainty in the estimates of traits [8,47].

Importantly for our approach here, while linguistic and plant-use borrowings to and from neighbouring cultures are not retrievable with PCMs, we can supplement and update our inferences with broader linguistic and historical analysis. For instance, our data suggest exchange of plant knowledge at extremely deep time-scales between North Germanic and Sámi (Uralic) populations, such as North Sámi *fádnu* (*fadno*) ‘angelica’ loaned from Proto-Norse *hwannu/Proto-Germanic *hwannō. The phonological form of North Sámi *fádnu* pre-dates a number of Proto-Norse (200–700 A.D.) sound changes (i.e. *a-*rounding via *u*-umlaut and *u-*elision, cf. Old Norse *hvo¸nn*), revealing the advanced age of this loan. Deeper historical linguistic analysis like this can complement PCM methods where we lack vertical signal in the data, distinguishing linguistic borrowings from inherited Norse-derived plant names. An example of a probable later borrowing is the transmission of medicinal plant uses from the Mediterranean tradition (fifth century BC to the nineteenth century [48]), which have been efficiently spread since the classical Greek herbals through multiple re-editions [48,49]. Such macroregional effects may explain the inferred medicinal uses of yarrow to treat digestive problems and injuries, uses already recommended by the Greco-Roman *materia medica* [50], the herbal medieval Nordic written tradition (electronic supplementary material, S4) and the Nordic modern oral knowledge (electronic supplementary material, S2). In sum, PCMs provide promising insights for vertically transmitted traits, but the broader historical and linguistic context must be iteratively deployed to corroborate or rule out some inferences.

Comparative analyses on relatively recent ethnographic or ethnobotanical data entail additional shortcomings. Idiosyncratic biases during data collection can result in only partly comparable datasets from different sites and authors [51]: for example, the veterinary plant uses collected here are mostly from Denmark and Norway and thus cannot be estimated as ancestral by our model, but surely existed throughout the Nordic region. Moreover, the method is not able to trace past uses that have been partly lost in modern times and thus not recorded in ethnobotanical sources, including some that must have been important—and even dominant—in certain periods of time (e.g. juniper as a construction material). This underlines the importance of triangulation with other existing sources of data. Another challenge stems from the etic categorization of traits from emic descriptions, resulting in potential problems with the reproducibility of coding [5]. Using standard classifications facilitates interoperability, but they are not a perfect match for every dataset [52]. Constraining the scope of analysis to a coherent cultural ‘clade’ as we have done here may mitigate these issues.

Finally, environmental constraints should be accommodated in PCM analyses [8,9]. Positive inference of ancestral plant uses will always be concomitant to the plant's presence in the environment—as endemic, cultivated or traded. Here, we are unable to reconstruct past plant uses of hops and woad that are evidenced through the archaeological context due to the limited geographical distribution of these plants. At present, controls for ecology can only be incorporated using multimodel inference when environmental factors such as plant areas of distribution are known (e.g. [53]); however, only language families, and not fine-grained phylogenetic relationships, can be represented in such models.

### (c) The added value of interdisciplinary collaboration

Triangulations made from archaeobotanical, historical and linguistic data, while much more desirable than inferences made solely with one of these data types, are still limited. Archaeobotanical data are informative about the presence of plants, but extremely rarely of their uses. Historical evidence is limited to cultures with a written tradition, texts are biased towards what was considered important to write down at a particular time [34], and they may be from a later time period than that of interest, as it is the case here. Linguistic evidence does not always allow precise dating and plant names that refer to uses should be analysed with care. Our study confirms that historical inferences made by using PCMs can be usefully added as a complimentary dataset to ‘triangulations’ that use data from anthropology, archaeology, philology and linguistics [32].

Interdisciplinary projects in cultural evolution face the challenges of arriving at shared conceptual references, vocabulary, data structures and research methods [1]. Our collaboration began with multidisciplinary discussion workshops to present and review the different potential sources of plant-use knowledge. We connected commonalities in data sources and methodologies across disciplines, discussed the scope and depth that each approach could contribute, and through continuous multidisciplinary dialogue, we strategically co-designed data collection of the independent datasets. Philological sources, plant names and archaeobotanical data were all systematically brought to bear on an interdisciplinary evaluation of PCM analyses on contemporary ethnobotanical data.

Collaborative interpretation of the results further allowed us to identify where PCMs inferred potentially ‘false positive’ ancestral uses [9], as well as the misidentification of botanical identities in historical documents. We might expect false positives when plants are widely cultivated (e.g. we would wrongly infer ancestral potato use in the Viking-Age), or when aspects of herbal traditions have been written down and profusely shared. Other than these easily identified cases, PCMs prove to be a very conservative method for estimating past plant uses: we observe that when a plant use is inferred to be ancestral, we find with high certainty that the plant was used in one way or another during the Viking-Age.

Identifying the role of plants in culture was the meeting point for our team of scholars in the humanities and the natural sciences. Issues of scale and focus are often considered to drive a wedge between these research traditions. For an historian, each source of plant knowledge should be studied and understood within the specific historical context. For an evolutionary biologist, the abstraction of details away from context in order to code knowledge in simple categories is necessary for computational analysis and generalization. In addressing ‘meso-evolutionary’ phenomena, we have treated different research approaches on equal terms, focusing on their complementarity. Our study also shows the value of a regional approach with a modest phylogeny: it allows thorough evaluation of the evidence from different disciplines for one research question.

## 5. Conclusion

We demonstrated a cultural evolutionary approach to understanding plant use through time in the Nordic regions. While ethnobotanical data are often used to infer plant remains in archaeological contexts, this is usually done arbitrarily. PCMs provide an additional toolkit with which to rationally weigh up how plants were used in pre-historic contexts. By using a phylogenetic comparative analysis with ethnobotanical data, we have inferred ancestral plant uses in the Viking-Age. We have triangulated and evaluated results against historical data from archaeobotanical, philological and linguistic datasets. The results of this interdisciplinary triangulation with archaeological and historical evidence confirm that PCMs can infer plant use, especially related to food and medicine, and the combination of all datasets suggests promising avenues for the study of plant use through time.

## Data accessibility

All data are provided as electronic supplementary material. The new code used in this article can be accessed here https://github.com/SimonGreenhill/noRdic.
