# Correction to ‘Historical, archaeological and linguistic evidence test the phylogenetic inference of Viking-Age plant use’

**DOI:** 10.1098/rstb.2022.0158

**Published:** 2022-06-20

**Authors:** Irene Teixidor-Toneu, Anneleen Kool, Simon J. Greenhill, Karoline Kjesrud, Jade J. Sandstedt, Vincent Manzanilla, Fiona M. Jordan

*Phil. Trans. R. Soc. B* **376**, 20200086. (Published 17 May 2021). (doi:10.1098/rstb.2020.0086)

Some funding information was inadvertently omitted from the funding statement.

This project has received funding from the European Union's Horizon 2020 research and innovation programme under the Marie Skłodowska-Curie grant agreement no. 841127.

